# Machine Learning Diagnostic Model for Early Stage NSTEMI: Using hs-cTnI 1/2h Changes and Multiple Cardiovascular Biomarkers

**DOI:** 10.3390/diagnostics14202322

**Published:** 2024-10-18

**Authors:** Junyi Wu, Yilin Ge, Ke Chen, Siyu Chen, Jiashu Yang, Hui Yuan

**Affiliations:** Department of Clinical Laboratory in Beijing Anzhen Hospital, Affiliated Hospital of Capital Medical University, Beijing 100029, China; wujunyi1688@mail.ccmu.edu.cn (J.W.); ge1017320015211@163.com (Y.G.); ckdexxy@163.com (K.C.); chens2001@163.com (S.C.); poplarsyang@163.com (J.Y.)

**Keywords:** NSTEMI, diagnostic model, machine learning

## Abstract

Background: This study demonstrates differences in the distribution of multiple cardiovascular biomarkers between non-ST-segment elevation myocardial infarction (NSTEMI) and unstable angina (UA) patients. Diagnostic machine learning predictive models measured at the time of admission and 1/2 h post-admission, achieving competitive diagnostic predictive results. Objective: This study aims to explore the diagnostic value of changes in high-sensitivity cardiac troponin I (hs-cTnI) levels in patients with suspected NSTEMI. Methods: A total of 267 patients presented with chest pain, requiring confirmation of acute coronary syndrome (ACS) subtypes (NSTEMI vs. UA). Hs-cTnI and other cardiac markers, such as creatine kinase-MB (CK-MB) and Myoglobin (Myo), were analyzed. Machine learning techniques were employed to assess the application of hs-cTnI level changes in the clinical diagnosis of NSTEMI. Results: Levels of CK-MB, Myo, hs-cTnI measured at admission, hs-cTnI measured 1–2 h after admission, and NT-proBNP in NSTEMI patients were significantly higher than those in UA patients (*p* < 0.001). There was a positive correlation between hs-cTnI and CK-MB, as well as Myo (R = 0.72, R = 0.51, R = 0.60). The optimal diagnostic model, Hybiome_1/2h, demonstrated an F1-Score of 0.74, an AUROC of 0.96, and an AP of 0.89. Conclusions: This study confirms the significant value of hs-cTnI as a sensitive marker of myocardial injury in the diagnosis of NSTEMI. Continuous monitoring of hs-cTnI levels enhances the accuracy of distinguishing NSTEMI from UA. The models indicate that the Hybiome hs-cTnI assays perform comparably well to the Beckman assays in predicting NSTEMI. Moreover, incorporating hs-cTnI measurements taken 1–2 h post-admission significantly enhances the model’s effectiveness.

## 1. Introduction

Acute coronary syndrome (ACS) encompasses a spectrum of clinical manifestations resulting from acute myocardial ischemia or necrosis, typically resulting from the complete or partial obstruction of a coronary artery by thrombosis [1]. This obstruction is frequently precipitated by the rupture or erosion of unstable atherosclerotic plaques. ACS is primarily classified into three distinct types: non-ST-segment elevation myocardial infarction (NSTEMI), ST-segment elevation myocardial infarction (STEMI), and unstable angina (UA) [2]. Suspected ACS or other acute chest pain symptoms account for a substantial portion of emergency department visits, comprising approximately 10% of all emergency cases [3].

When ACS is suspected, current clinical guidelines recommend performing an ECG within 10 min [4]. If ST-segment elevation is detected, coronary angiography (CAG) should be performed within 2 h for definitive diagnosis and treatment of STEMI. If ACS symptoms are present without ST-segment elevation, high-sensitivity cardiac troponin T or I (hs-cTnT or hs-cTnI) should be measured within 3 h. If the initial test results are normal, a repeat test should be conducted 1–2 h later [5]. ACS can be reliably excluded with a negative predictive value of approximately 99% following two consecutive negative high-sensitivity cardiac troponin tests. On the other hand, a significant elevation in high-sensitivity troponin levels may indicate NSTEMI, and CAG will be promptly performed to assess obstruction.

Hs-cTn, while highly sensitive, are often criticized for their low specificity [6]. Although they can be used to detect very low levels of cardiac troponins, making them useful for early detection of myocardial injury, elevated hs-cTn levels can also be observed in non-cardiac conditions such as renal failure, sepsis, pulmonary embolism, or even strenuous exercise. This lack of specificity can lead to false positives, complicating the diagnosis of ACS and increasing the risk of unnecessary investigations or treatments [7]. This study aims to utilize machine learning methods, integrating changes in hs-cTnI levels along with various other clinical biomarkers, to develop a combined predictive model that assists in the diagnosis of NSTEMI and UA. The goal is to diminish superfluous invasive CAG.

The widespread use of hs-cTn has significantly improved the sensitivity of acute chest pain diagnosis [8,9,10]. The 0/1h and 0/2h hs-cTnI measurement protocols, as well as relative changes in hs-cTnI levels, have already been applied in the clinical diagnosis of acute myocardial infarction (AMI) [5,11,12]. These rapid assessment algorithms allow for earlier detection or exclusion of AMI by monitoring changes in troponin levels within a short time frame, thus facilitating timely decision-making and reducing the need for prolonged observation or unnecessary invasive procedures. In this paper, we used the 0/2h hs-cTnI levels and other clinical biomarkers to develop a diagnostic model for NSTEMI and UA using LightGBM. This approach aims to enhance diagnostic accuracy and provide a more efficient tool for distinguishing between these conditions before invasive CAG.

Machine learning has been widely applied in clinical diagnostics [13,14,15,16]. LightGBM [17,18] is a decision tree-based gradient-boosting framework developed by Microsoft. Compared to other commonly used models such as Random Forest, XGBoost [19], or neural networks, LightGBM offers several significant advantages, making it the optimal choice for this study. First, LightGBM is known for its fast training speed, which is crucial for handling large-scale, high-dimensional data. Additionally, LightGBM uses a histogram-based algorithm that groups continuous features into bins, reducing computational complexity and speeding up training. This approach improves efficiency in handling imbalanced datasets and strengthens diagnostic model robustness. In comparison, XGBoost, while powerful, often has slower training times and higher memory usage on large datasets. Neural networks, though good at capturing complex non-linear patterns, generally need longer training times, more complex designs, and hyperparameter tuning. They also have lower interpretability, making them less ideal for tasks requiring clear diagnostic reasoning.

In comparison, LightGBM maintains high performance while offering superior interpretability, particularly in scenarios where it is necessary to explain the contribution of each feature to the model’s predictions. Its feature importance functionality enables researchers to better understand the model’s decision-making process, thereby increasing trust in applications such as the medical field. For instance, studies such as those by [20,21,22] have employed LightGBM as the optimal diagnostic model.

The proposed LightGBM model is built using 0/2h hs-cTnI levels and other biomarkers as features, aiming to diagnose NSTEMI and UA before invasive coronary angiography. The main contributions of this paper are as follows:

1. We developed a LightGBM model using hs-cTnI levels at admission and after 2 h, as well as other biomarkers, achieving competitive results in predicting and diagnosing NSTEMI and UA.
2. Through experiments, we demonstrated that incorporating hs-cTnI levels at admission and after 2 h significantly improved the model’s predictive performance.
3. We conducted a comparative analysis to assess the impact of different hs-cTnI measurement systems and reagents from two companies on the model’s predictive capability.

## 2. Materials and Methods

### 2.1. Study Subjects

This study included 267 patients who presented with chest pain and required differentiation of ACS subtypes at the Emergency Center of Beijing Anzhen Hospital, Capital Medical University, between March 2023 and February 2024. Based on comprehensive clinical evaluations, these patients were classified into two groups: NSTEMI and UA. Cardiologists determine the final diagnoses by thoroughly analyzing the patient’s medical histories, physical examinations, laboratory tests, echocardiography, and imaging results, ensuring both diagnostic accuracy and comprehensiveness.

Inclusion criteria were Chinese patients aged 18 years and older who experienced chest pain, angina, or other symptoms indicative of myocardial ischemia (e.g., shortness of breath, nausea, vomiting, sweating, fatigue) within 12 h, consistent with NSTEMI. Exclusion criteria included patients with a confirmed diagnosis of STEMI (characterized by significant ST-segment changes or T-wave inversion on ECG), those with renal insufficiency requiring dialysis, patients in the active phase of an autoimmune disease, pregnant or cancer patients, and those with severe skeletal muscle injury.

### 2.2. Sample Collection and Testing

Hs-cTnI, recommended by the European Society of Cardiology (ESC) as the primary biomarker as it is the most accurate indicator of ACS, will be tested at two time points using two different reagents [23,24,25]. Myoglobin elevation can result from AMI, as it is usually used alongside more specific cardiac biomarkers, such as hs-cTnI, to improve the accuracy of early AMI diagnosis [26]. Creatine kinase-MB (CK-MB) is a well-established biomarker for diagnosing AMI [27]. NT-proBNP is a valuable adjunct in diagnosing ACS and NSTEMI, providing critical insights into cardiac function. Elevated NT-proBNP levels during the diagnostic evaluation of ACS [28]. Therefore, the mentioned biomarkers were chosen for testing.

Two 5 mL samples of pro-coagulant venous blood were collected from each patient: the first sample was obtained immediately upon admission, and the second 1–2 h later. According to the American Association for Clinical Chemistry (AACC) Academy Laboratory Medicine Practice Guidelines [29], repeating the blood test to measure hs-cTnI 1–2 h after the initial test can confirm changes in troponin levels. Both blood samples were centrifuged at 2000 rpm for 8 min to separate the serum. The separated serum was divided into two portions: one portion was immediately used to quantify hs-cTnI concentration using the UniCel DXI 800 Access system (Beckman Coulter, Brea, CA, USA) and its reagents, while the other portion was used to measure NT-proBNP and hs-cTnI levels using the Hybiome AE180 chemiluminescent immunoassay analyzer (Hybiome, Suzhou, China) and its reagents. CK-MB and Myo concentrations were measured in both portions.

### 2.3. Statistical Analysis

Data analysis was conducted in Python 3.10, covering statistical and correlation analyses. A baseline table summarized the study population’s demographic and clinical characteristics. Continuous variables were reported as mean ± SD or median (IQR) based on data distribution, while categorical variables were shown as percentages. Group comparisons used the Wilcoxon rank sum test or Welch Two Sample *t*-test for continuous variables and the Chi-Square test for categorical variables. Statistical significance was set at a two-tailed *p*-value < 0.05. Pearson’s correlation coefficients were calculated to examine relationships between key variables (Myo\CK-MB\hs-cTnI\NT-proBNP) based on data normality.

### 2.4. Machine Learning Methods

#### 2.4.1. Data and Model Design

The dataset comprises 267 samples, including 36 NSTEMI and 231 UA cases, leading to a highly imbalanced class distribution. This imbalance is significant because it can affect diagnostic accuracy, necessitating careful model selection and evaluation. We assessed two key factors: (1) the impact of the detection reagent on diagnostic outcomes, and (2) the contribution of hs-cTnI measurements taken 1–2 h post-admission to diagnostic accuracy.

We developed several diagnostic models using different biomarkers and measurement systems. The Beckman_0h model utilized hs-cTnI concentrations measured at admission using the Beckman system and reagents. The Hybiome_0h model incorporated NT-proBNP and hs-cTnI concentrations measured at admission using the Hybiome system and reagents. Both models also included CK-MB and Myo levels measured at admission. The Beckman_1/2h model and the Hybiome_1/2h model employed the same detection methods to measure the concentrations of these biomarkers again 1–2 h after admission.

All four experiments used identical data preprocessing and hyperparameter search techniques [30]. The experiments were conducted in Python 3.10, utilizing Sklearn and LightGBM for machine learning, as well as Pandas, Numpy, and SMOTE for data processing and visualization. The code and data are available from the corresponding author upon request.

#### 2.4.2. Model Validation

To address the challenge of imbalanced class distribution, we employed 3-fold cross-validation, which enhances model stability by ensuring a greater representation of positive samples in each validation set. Patients with NSTEMI and UA were divided into a training set (67%) and a validating set (33%) for the development and validation of the decision tree model. 

In the training set, we applied the Borderline-SMOTE data augmentation method, a technique specifically designed to improve the representation of minority classes near decision boundaries. While SMOTE generally mitigates data imbalance by generating new minority class samples [31], Borderline-SMOTE further refines this process by creating synthetic samples through linear interpolation between boundary samples and their neighbors [32]. Given our focus on diagnosing a limited number of positive NSTEMI cases, the application of Borderline-SMOTE significantly enhanced the model’s recall rate, thereby improving overall diagnostic accuracy.

The model’s performance was evaluated using three key metrics: AUROC, F1-score, and AP. AUROC assesses the model’s ability to distinguish between positive and negative samples and is particularly useful for balanced class distributions. The F1-score, the harmonic mean of Precision and Recall, is well-suited for evaluating classification performance in imbalanced datasets, as it emphasizes the model’s accuracy in identifying positive samples. AP, which measures the area under the Precision–Recall (PR) curve, evaluates the model’s ability to predict positive samples across various thresholds and provides an average Precision value, highlighting performance at different levels of Recall [33].

Many studies have focused on the clinical interpretability of machine learning models, such as [14,34]. We used the SHapley Additive exPlanation (SHAP) algorithm to evaluate the feature importance of our model [34]. SHAP values provide a measure of each feature’s contribution to the model’s predictions, allowing for a more interpretable understanding of how individual features impact the output of the model. SHAP calculates feature importance by considering the marginal contribution of each feature across all possible combinations of features. This approach is grounded in Shapley values from cooperative game theory, ensuring a fair distribution of feature importance regardless of the order in which features are introduced [35]. A higher positive SHAP value indicates that the feature contributes to increasing the model’s prediction, while a negative SHAP value suggests that the feature drives the prediction downward.

Given our uneven sample distribution, we prioritized the AP value, as it best reflects the model’s effectiveness in handling imbalanced classes. To optimize the model, we used the TPE Bayesian optimization algorithm over 500 iterations, with AP as the objective function.

## 3. Results

### 3.1. The Distribution of Clinical Characteristics

#### 3.1.1. Demographic and Clinical Characteristics

Table 1 presents the clinical characteristics of 267 patients, with 231 (87%) in the UA group and 36 (13%) in the NSTEMI group. The median ages were similar between the groups (UA: 61 years, NSTEMI: 63.5 years). There were no significant differences in systolic or diastolic blood pressure at admission (SBP: 149 mmHg vs. 142.5 mmHg; DBP: 82.71 mmHg vs. 84.86 mmHg). The gender distribution was also comparable across groups, with 56.93% of patients being male (UA: 56.28%, NSTEMI: 61.11%). Additionally, no significant differences were observed in the prevalence of hypertension (*p* = 0.059), diabetes (*p* = 0.130), or prior cardiac surgery (*p* = 0.071). However, coronary heart disease was significantly more common in the NSTEMI group (69.44% vs. 40.69%; *p* < 0.001). Overall, the demographic and clinical characteristics were largely comparable between the two groups.

#### 3.1.2. Cardiac Biomarkers

Cardiac biomarkers significantly differed between the NSTEMI and UA groups, which is also indicated in Table 1. At admission, myoglobin levels were higher in the NSTEMI group (36.50 ng/mL) compared to the UA group (20.80 ng/mL) (*p* < 0.001). Similarly, CK-MB levels were elevated in the NSTEMI group (6.15 ng/mL) versus the UA group (1.30 ng/mL) (*p* < 0.001). Hs-cTnI levels at both T0 and T1-2 hours were significantly higher in the NSTEMI group. Using the Beckman assay, median hs-cTnI levels at T0 were 403.35 ng/L for NSTEMI and 3.00 ng/L for UA (*p* < 0.001). At T1-2 hours, the levels were 683.55 ng/L for NSTEMI and 2.70 ng/L for UA (*p* < 0.001). The Hybiome assay also showed significant differences in hs-cTnI between groups at both time points (*p* < 0.001). Additionally, NT-proBNP levels were markedly higher at admission in the NSTEMI group (median 1392.55 pg/mL) compared to the UA group (median 104.76 pg/mL, *p* < 0.001).

### 3.2. Correlation Analysis of Cardiac Biomarkers

**Figure 1:**
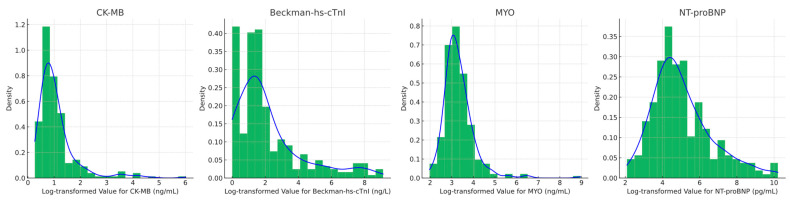
Distribution of log-transformed biomarker values at admission. Histograms represent the log-transformed values of the cardiac biomarkers CK-MB, Beckman-hs-cTnI, Myoglobin (MYO), and NT-proBNP measured at admission. The blue density line overlays the histogram, illustrating the probability distribution of each biomarker. The x-axis represents the log-transformed values, while the y-axis indicates the density.

**Figure 2:**
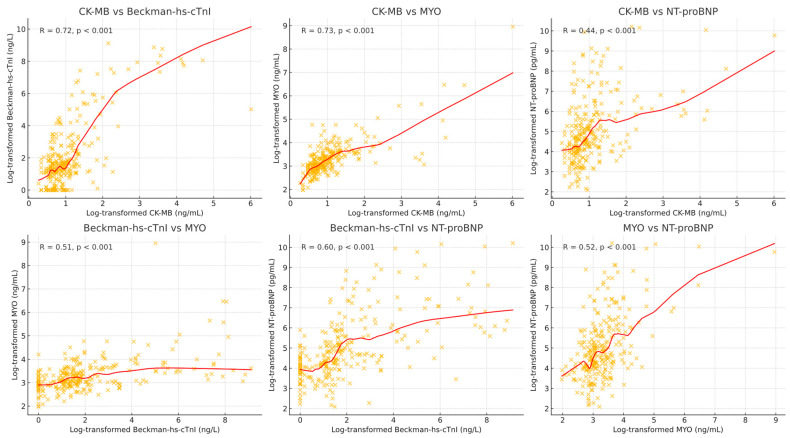
Correlation plots between log-transformed cardiac biomarker values at admission. Scatter plots display pairwise correlations between the log-transformed values of CK-MB, Beckman-hs-cTnI, Myoglobin(MYO), and NT-proBNP at admission. The red line represents a locally weighted scatterplot smoothing (LOESS) fit, while the R values and p-values indicate the strength and significance of the correlation. Each plot shows a distinct relationship between two biomarkers, highlighting both linear and non-linear patterns of association across the dataset.

A correlation analysis of CK-MB, myoglobin, hs-cTnI, and NT-proBNP levels at admission was conducted on 267 patients. Figure 1 presents the distribution of log-transformed biomarker values at admission. Figure 2 presents the correlation plots between log-transformed cardiac biomarker values using scatter plots, and correlation coefficients to illustrate the relationships among these cardiac biomarkers. These two figures highlight the linear relationships and distributions of the biomarkers, providing valuable insights for cardiovascular research. Notably, there is a strong positive correlation between CK-MB and myoglobin levels at admission (Pearson R = 0.73, *p* < 0.001). Hs-cTnI levels at admission show a moderate correlation with NT-proBNP (R = 0.60, *p* < 0.001), a strong correlation with CK-MB (R = 0.72, *p* < 0.001), and a low correlation with NT-proBNP (R = 0.44, *p* < 0.001).

### 3.3. Construction of the NSTEMI Diagnostic Model

**Figure 3:**
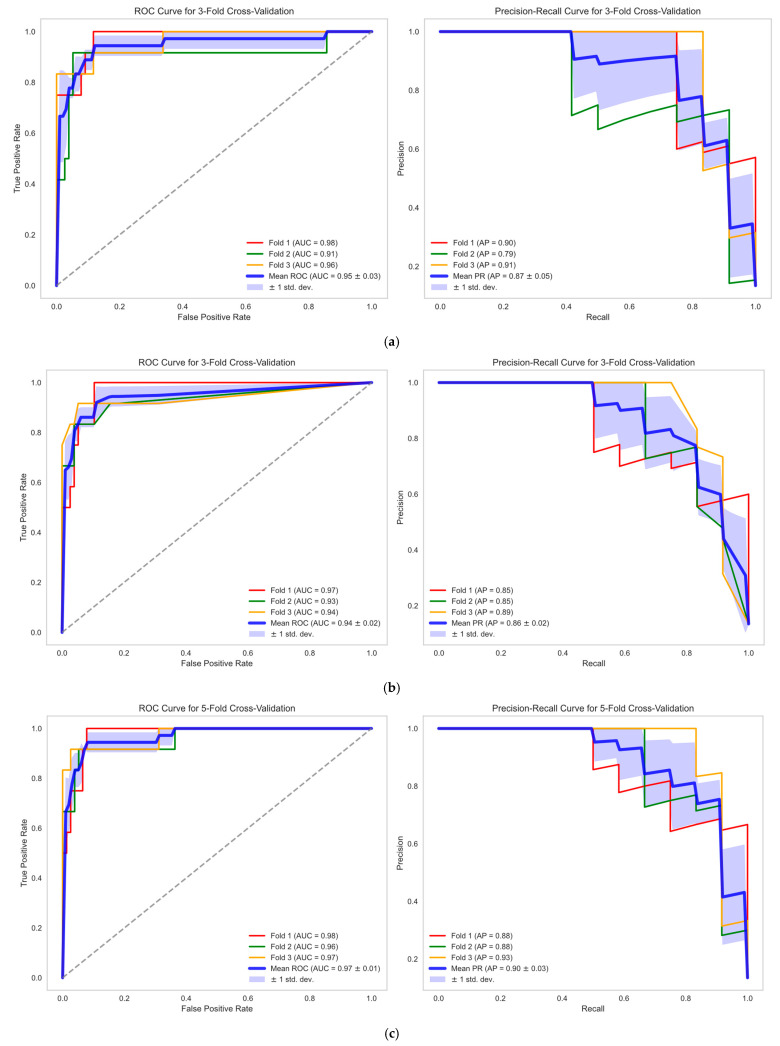

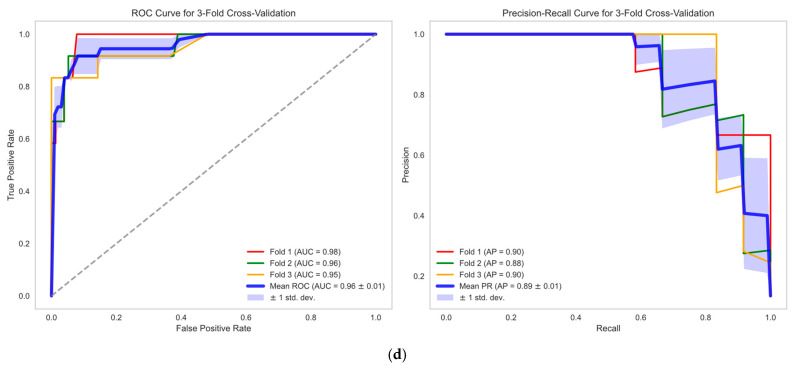
ROC and Precision–Recall curves for 3-fold cross-validation across (**a**) Beckman_0h, (**b**) Hybiome_0h, (**c**) Beckman_1/2h, and (**d**) Hybiome_1/2h models. ROC Curves (left panels): The blue line represents the mean ROC curve across the three folds, while the shaded area around the mean curve denotes the standard deviation (SD). This highlights the model’s consistency and reliability in distinguishing between classes across different subsets of data. The Area Under the Curve (AUC) values are also provided for each fold, with the overall mean AUC and its SD indicated, demonstrating the model’s discriminative power. Precision–Recall Curves (Right Panels): Like the ROC curves, the mean Precision–Recall curve is illustrated with a blue line, and the shaded region represents the standard deviation (SD). The Average Precision (AP) values for each fold are provided, alongside the mean AP and its SD.

In this study, we evaluated the classification performance of models under different experimental conditions, including Beckman_0h, Hybiome_0h, Beckman_1/2h, and Hybiome_1/2h. The Precision, Recall, F1-Score, AUROC, and AP metrics for each condition are summarized in Table 2. The detailed ROC and PR curves for the four models in the three-fold cross-validation are shown in Figure 3.

The Beckman_0h model achieved an F1-Score of 0.71, an AUROC of 0.95, and an AP of 0.87, indicating strong performance with high precision and effective differentiation capability for the Beckman system and its corresponding reagents. The Hybiome_0h model demonstrated a significantly improved F1-Score of 0.79, while the AUROC slightly decreased to 0.95, and the AP remained stable at 0.86. These results suggest that the Hybiome_0h model outperformed the Beckman_0h model, particularly in terms of F1-Score.

Under Beckman_1/2h conditions, the model achieved an F1-Score of 0.76. Notably, the AUROC significantly increased to 0.97, and the AP improved to 0.90, demonstrating the model’s enhanced discrimination ability. These results underscore the benefit of remeasuring hs-cTnI levels one to two hours after admission, which significantly aids in distinguishing between NSTEMI and UA. The Hybiome_1/2h model achieved an F1-Score of 0.74. Despite a slight decrease in Recall, the model maintained a high AUROC of 0.96 and an AP of 0.89, indicating strong overall performance.

The 1/2h models, particularly under Beckman_1/2h conditions, demonstrated superior performance with the highest AUROC and AP values, emphasizing their strength in differentiating between classes. These findings suggest that remeasuring hs-cTnI levels 1–2 h after patient admission is beneficial for diagnosing NSTEMI and UA.

Moreover, the Hybiome_1/2h model, despite a minor decrease in Recall, showed performance very close to that of the Beckman_1/2h model, particularly in terms of Precision, AUROC, and AP. This suggests that both the Hybiome and Beckman 1/2h models are highly effective, with Beckman_1/2h having a slight advantage, while Hybiome remains a competitive alternative.

We also conducted an ablation study comparing the use and non-use of Borderline-SMOTE, with the results presented in Table 3. These results demonstrate that Borderline-SMOTE data augmentation enhances the model’s predictive capabilities on this dataset.

### 3.4. Feature Importance Analysis

**Figure 4:**
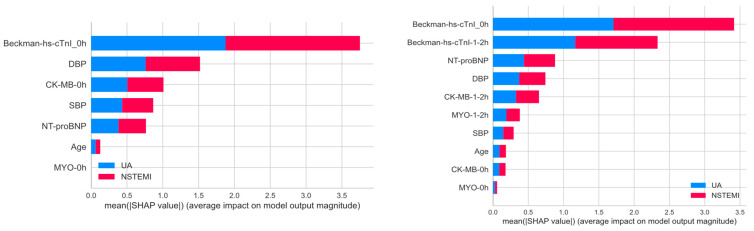
The bar plots display the mean absolute SHAP values of features calculated from models trained using clinical data to predict outcomes of UA and NSTEMI. The left plot represents the feature importance for the Beckman_0h model, while the right plot corresponds to the Beckman_1/2h model. Blue bars indicate the contribution to predicting UA, and red bars show the contribution to predicting NSTEMI.

We also analyzed the feature importance of the Beckman_0h and Beckman_1/2h models, as shown in Figure 4. From the SHAP bar plots, we can conclude the following:

The hs-cTnI levels at admission consistently emerge as the most significant feature in both models, with a stronger impact on predicting NSTEMI. In the Beckman_1/2h model, the importance of hs-cTnI levels after 1 or 2 h post-admission increases significantly, particularly for NSTEMI, suggesting that changes in hs-cTnI levels over time contribute significantly to the model’s predictions as well.

Other features, such as DBP, CK-MB, SBP, and NT-proBNP levels, show varying degrees of importance and contribute to the predictions of both UA and NSTEMI to a certain extent.

## 4. Discussion

This study performed an in-depth analysis of biomarker data from patients with UA and NSTEMI using both statistical and machine-learning approaches. Our results showed that hs-cTnI levels were significantly elevated in the NSTEMI group, consistent with the existing literature, which identifies hs-cTnI as a highly sensitive marker of myocardial injury. Furthermore, we developed a LightGBM model incorporating hs-cTnI measurements at admission and after 1–2 h, along with other relevant biomarkers, achieving strong predictive and diagnostic performance for both NSTEMI and UA. Our experiments demonstrated that integrating both the hs-cTnI levels at admission and after 1–2 h significantly enhanced the model’s accuracy. Additionally, we conducted a comparative analysis to evaluate the influence of different hs-cTnI measurement systems and reagents from two manufacturers on the model’s predictive capability, providing insights into potential variations in diagnostic outcomes.

Our findings have significant implications for clinical practice. First, when the clinical symptoms of UA and NSTEMI are quite similar, we found that patients with a history of heart disease are more likely to have NSTEMI, and a series of cardiac biomarkers will be different, including the common Myoglobin, CK-MB, hs-cTnI, NT-proBNP(*p* < 0.001). Second, our work emphasized the importance of continuous monitoring of hs-cTnI changes in 1–2 h in diagnosing NSTEMI patients. Second, the developed algorithm model in our research may help optimize the combined batch of biomarkers to enhance the diagnostic accuracy of NSTEMI. These results have practical significance for real-world clinical applications.

Diagnosing early-stage NSTEMI is particularly challenging because many patients do not exhibit typical symptoms or specific changes on ECGs [36]. In clinical practice, the combined early elevation of Myoglobin, CK-MB, and the high sensitivity of hs-cTnI are often used as a multi-marker strategy for diagnosing AMI [37]. This comprehensive strategy enhances the accuracy and timeliness of MI diagnosis. Additionally, the multi-marker approach improves risk stratification, informs clinical management decisions, and allows for effective monitoring of therapeutic outcomes in patients with ACS [38]. Our experiments confirmed the significance of using a combination of multiple biomarkers, and applying the LightGBM algorithm resulted in higher accuracy.

However, our study has certain limitations. While the results offer insights into the interactions between biomarkers and their diagnostic capabilities, these analyses cannot establish causal relationships. Additionally, the presence of outliers highlights the need for more refined methods to better understand the differences in biomarker levels among individual patients. The study was also constrained by a relatively small sample size and significant class imbalance, which affected the generalization ability of the trained models. Future research should consider larger sample sizes and incorporate additional clinical factors to further validate and refine these findings.

In conclusion, this study provides new insights into the correlation and variability of biomarkers in the diagnosis of NSTEMI and demonstrates the potential of multivariate statistical methods in diagnosing NSTEMI patients.

## 5. Conclusions

This study demonstrates differences in the distribution of multiple cardiovascular biomarkers between NSTEMI and UA patients. We constructed diagnostic machine learning predictive models using hs-cTnI and other cardiovascular biomarkers measured at the time of admission and 1–2 h post-admission, achieving competitive diagnostic predictive results. Specifically, comparing the Beckman_1/2h model to the Beckman_0h model, we observed performance improvements: accuracy increased by 3.33%, precision by 11.48%, recall by 3.49%, F1 score by 7.04%, AUROC by 2.11%, and AP by 3.45%. These findings contribute to the development of early diagnostic models for NSTEMI, helping to diminish superfluous invasive treatments such as CAG. We believe that with larger datasets and improved predictive techniques, NSTEMI diagnosis can become more accurate through the use of clinical biomarkers, enabling earlier intervention and minimizing the over-reliance on invasive methods.

## Figures and Tables

**Table 1:** Clinical characteristics and cardiac biomarkers distribution in NSTEMI vs. UA patients.

Variable		Diagnosis		
	Overall, *n* = 267 ¹	UA *n* = 231(87%) ¹	NSTEMI *n* = 36(13%) ¹	*p*-Value ²
Age	61.00[50.50,68.50]	61.00[50.00,68.00]	63.50[55.50,71.00]	
SBP	149.00[134.50,165.00]	149.00[135.00,165.00]	142.50[129.24,161.50]	
DBP	83.00[13.22]	82.71[12.52]	84.86[17.13]	
Gender				
Male	152 (56.93%)	130(56.28%)	22(61.11%)	
Female	22(61.11%)	101(43.72%)	14(39.89%)	
History				
Hypertension	143(53.56%)	126(54.55%)	17(47.22%)	
Diabetes mellitus	41(15.36%)	33(14.29%)	8(22.22%)	
Cardiac heart disease	119(44.57%)	94(40.69%)	25(69.44%)	<0.001
Cardiac surgery	48(17.98%)	35(15.15%)	13(36.11%)	
Cardiac biomarkers				
Myoglobin(Beckman assay)	22.55[17.55,5.30]	20.80[16.80,31.50]	36.50[30.03,102.80]	<0.001
CK-MB(Beckman assay)	1.40[0.90,2.30]	1.30[0.90,1.90]	6.15[2.78,29.35]	<0.001
Hs-cTnI_0h(Beckman assay)	3.70[1.50,14.80]	3.00[1.10,6.20]	403.35[139.85,2386.65]	<0.001
Hs-cTnI_1/2h(Beckman assay)	3.80[1.30,18.15]	2.70[0.90,6.05]	683.55[167.48,2604.70]	<0.001
Hs-cTnI_0h(Hybiome assay)	7.56[4.13,30.94]	6.40[3.87,12.12]	914.90[228.09,4470.67]	<0.001
Hs-cTnI_1/2h(Hybiome assay)	7.76[4.01,32.74]	6.25[3.57,15.97]	1593.37[356.00,5610.44]	<0.001
NT-proBNP(Hybiome assay)	107.78[54.05,349.18]	104.76[43.67,315.06]	1392.55[319.43,3348.76]	<0.001

^1^ Median [IQR]; Mean (SD); *n*(%). ^2^ Wilcoxon rank sum test; Welch Two Sample *t*-test; Chi-squared test.

**Table 2:** Performance metrics of various experiments on model performance.

Experiment	Accuracy	Precision	Recall	F1	AUROC	AP
Beckman_0h	0.90	0.61	0.86	0.71	0.95	0.87
Hybiome_0h	0.94	0.73	0.89	0.79	0.95	0.86
Beckman_1/2h	0.93	0.68	0.89	0.76	0.97	0.90
Hybiome_1/2h	0.93	0.71	0.78	0.74	0.96	0.89

**Table 3:** Ablation study comparing the effects of Borderline-SMOTE versus no SMOTE across four different models.

Models	Method	Accuracy	Precision	Recall	F1	AUROC	AP
Beckman_0h	No SMOTE	0.93	0.75	0.75	0.75	0.96	0.86
	Borderline SMOTE	0.90	0.61	0.86	0.71	0.95	0.87
Hybiome_0h	No SMOTE	0.91	0.62	0.81	0.70	0.95	0.85
	Borderline SMOTE	0.94	0.73	0.89	0.79	0.95	0.86
Beckman_1/2h	No SMOTE	0.92	0.68	0.83	0.75	0.97	0.89
	Borderline SMOTE	0.93	0.68	0.89	0.76	0.97	0.90
Hybiome_1/2h	No SMOTE	0.92	0.69	0.75	0.72	0.97	0.89
	Borderline SMOTE	0.93	0.71	0.78	0.74	0.96	0.89

## Data Availability

The original contributions presented in the study are included in the article, further inquiries can be directed to the corresponding author.
